# PU.1 is required to restrain myelopoiesis during chronic inflammatory stress

**DOI:** 10.3389/fcell.2023.1204160

**Published:** 2023-06-26

**Authors:** James S. Chavez, Jennifer L. Rabe, Katia E. Niño, Harrison H. Wells, Rachel L. Gessner, Taylor S. Mills, Giovanny Hernandez, Eric M. Pietras

**Affiliations:** ^1^ Division of Hematology, University of Colorado Anschutz Medical Campus, Aurora, CO, United States; ^2^ Department of Immunology and Microbiology, University of Colorado Anschutz Medical Campus, Aurora, CO, United States

**Keywords:** hematopoiesis, inflammation, myelopoiesis, PU.1, hematopoietic stem cell, hematopoietic progenitor cell

## Abstract

Chronic inflammation is a common feature of aging and numerous diseases such as diabetes, obesity, and autoimmune syndromes and has been linked to the development of hematological malignancy. Blood-forming hematopoietic stem cells (HSC) can contribute to these diseases via the production of tissue-damaging myeloid cells and/or the acquisition of mutations in epigenetic and transcriptional regulators that initiate evolution toward leukemogenesis. We previously showed that the myeloid “master regulator” transcription factor PU.1 is robustly induced in HSC by pro-inflammatory cytokines such as interleukin (IL)-1β and limits their proliferative activity. Here, we used a PU.1-deficient mouse model to investigate the broader role of PU.1 in regulating hematopoietic activity in response to chronic inflammatory challenges. We found that PU.1 is critical in restraining inflammatory myelopoiesis via suppression of cell cycle and self-renewal gene programs in myeloid-biased multipotent progenitor (MPP) cells. Our data show that while PU.1 functions as a key driver of myeloid differentiation, it plays an equally critical role in tailoring hematopoietic responses to inflammatory stimuli while limiting expansion and self-renewal gene expression in MPPs. These data identify PU.1 as a key regulator of “emergency” myelopoiesis relevant to inflammatory disease and leukemogenesis.

## Introduction

Chronic inflammation is a widespread physiological consequence of aging and physiological decline and is likewise associated with a broad range of disease states, including autoimmune disease, diabetes, and obesity (Campisi, 2013; Jaiswal, 2020). These phenotypes are often characterized by the overproduction of myeloid cells that infiltrate into diseased or damaged tissues, thereby contributing to disease pathogenesis. Chronic inflammation has also been linked to the development and/or progression of various cancers (Laconi et al., 2020; Marongiu and DeGregori, 2022). This includes myeloid malignancies like myelodysplastic syndrome (MDS) (Ganan-Gomez et al., 2015; Zambetti et al., 2016; Barreyro et al., 2018; Trowbridge and Starczynowski, 2021) and acute myelogenous leukemia (AML) (Carey et al., 2017; Chakraborty et al., 2021). This wide spectrum of immunological and malignant diseases may trace its origin to the activation of blood-forming hematopoietic stem cells (HSC) by inflammatory signals (Ganan-Gomez et al., 2015; Muto et al., 2020; Caiado et al., 2021; Chakraborty et al., 2021; Higa et al., 2021; Florez et al., 2022; Weeks et al., 2022). Understanding the mechanism(s) by which expansion and production of hematopoietic stem and progenitor cells (HSPC) and their myeloid progeny are regulated in chronic inflammation is critical to establishing more effective interventions that reduce pathologies associated with inflammatory disease and limit the risk of initiating hematological malignancy.

We and others have previously shown that the myeloid ‘master regulator’ transcription factor PU.1 (DeKoter et al., 1998; Singh et al., 1999) is upregulated at the transcriptional and protein levels in HSC by chronic inflammatory signals such as the pro-inflammatory cytokine IL-1β (Algeciras-Schimnich et al., 2003; Pietras et al., 2016; Hernandez et al., 2020; Rabe et al., 2020; Chavez et al., 2021; Ahmed et al., 2022; Chavez et al., 2022). IL-1β is produced in response to a wide range of physiological insults, and IL-1 signaling is closely linked to a wide variety of chronic inflammatory diseases, where it plays a critical role in the activation of inflammatory myelopoiesis (Ueda et al., 2009; Dinarello, 2011; Mirantes et al., 2014; Pietras, 2017). Using a mouse model of chronic rheumatoid arthritis, we previously showed that myeloid cell production and accompanying PU.1-driven myeloid gene programs in HSC can be blocked pharmacologically using the IL-1 receptor (IL-1R) antagonist anakinra (Hernandez et al., 2020). IL-1 signaling is also linked to somatic evolutionary processes that give rise to myeloid malignancy (Barreyro et al., 2012; Pietras et al., 2016; de Mooij et al., 2017; Caiado et al., 2021; Burns et al., 2022; Caiado et al., 2022). In phenotypically defined long-term HSC, increased PU.1 levels drive 1) activation of myeloid differentiation gene programs and 2) repression of cell proliferation (Chavez et al., 2021), thereby limiting the expansion of the HSC pool. However, these two functions of PU.1 may appear at odds given the classical understanding of PU.1 function is to facilitate myelopoiesis. Notably, loss of PU.1 activity is closely associated with myeloid leukemia, and the PU.1 network is commonly disrupted in myeloid hematological malignancies, though PU.1 itself is rarely mutated (Will et al., 2015; Aivalioti et al., 2022). While complete genetic ablation of PU.1 can yield a profound differentiation block that graduates to an AML-like phenotype (Steidl et al., 2006), early stages of myeloid oncogenesis are typically characterized by graded reductions in PU.1 activity due to the action of oncogenic mutations, rather than complete ablation of expression (Will et al., 2015). However, the extent to which loss of PU.1 function impacts myelopoiesis in response to chronic inflammation has not been investigated.

Here, our study aimed to evaluate the role of PU.1 in regulating hematopoietic responses to chronic inflammation, to better understand how the distinct functions of PU.1 (cell cycle regulation, myeloid differentiation) intersect to regulate myeloid output and the characteristic expansion of myeloid progenitors that occurs in this context. To address these questions, we employed the PU.1 knock-in (KI) mouse model, in which a deactivating point mutation was knocked into the PU.1 autoregulatory binding site within the −14 kb upstream regulatory element (URE), leading to graded loss of PU.1 function without the development of overt leukemia-like disease (Staber et al., 2013). During chronic *in vivo* IL-1β stimulation, we find that PU.1 is required to suppress excess myeloid cell production and properly regulate the balance between differentiation, self-renewal, and proliferation in HSPC populations following chronic inflammatory challenge.

## Materials and methods

### Mice

*PU.1* ^*KI/KI*^ mice were provided as a kind gift by Dr. Dan Tenen (Harvard Stem Cell Institute). *PU.1* ^*KI/KI*^ mice were bred to C57BL/6J mice (strain #000664) from The Jackson Laboratory to generate *PU.1* ^*+/+*^ littermate controls for the study. 6–12-week-old animals were used for experiments, and animals of both sexes were used in these studies. Mice were euthanized by CO_2_ inhalation followed by cervical dislocation. All animal experiments and euthanasia procedures were conducted in accordance with approved procedures reviewed by the Institutional Animal Care and Use Committee (IACUC) at University of Colorado Anschutz Medical Campus (protocol number 00091).

### *In vivo* studies

0.5 μg of IL-1β (Peprotech) suspended in sterile D-PBS/0.2% BSA, or D-PBS/0.2% BSA alone as a -IL-1β control, was injected intraperitoneally (i.p.) in a 100 μL bolus daily for 20 days, as previously described in the literature. *In vivo* puromycin incorporation assays were performed by injecting 500 μg puromycin in a 100 μL bolus intraperitoneally 1 h prior to euthanasia, following previously published protocols.

### Flow cytometry

For analysis of BM cell populations, we used an identical protocol as our previously published work. BM was flushed from the four long bones (femurs + tibiae) of mice using a syringe and 21G needle filled with staining media (SM: 2% heat-inactivated FBS in HBSS without Ca^2+^ or Mg^2+^). Cells were subsequently pelleted at 500 x g and resuspended in 1x ACK (ACK 150 mM NH_4_Cl/10 mM KHCO_3_) on ice for 3–5 min to deplete red blood cells prior to washing with SM and filtering through a 70 micron nylon mesh to remove debris. Total cell numbers were determined using a ViCell automated counter (Beckman-Coulter) and 1 × 10^7^ BM cells were used for staining. To identify HSPC populations, BM cells were stained for 30 min on ice with PE-Cy5-conjugated anti-CD3, CD4, CD5, CD8, Gr-1 and Ter119 to exclude mature lineage + cells, plus Flk2-biotin, Mac-1-PE/Cy7, FcγR-APC, CD48-A700, and cKit-APC/Cy7. Purified rat IgG was also included as a blocking agent. Following a wash step, BM cells were stained with Sca-1-BV421, CD41-BV510, CD150-BV785, and streptavidin (SA)-BV605 in SM with a 1:4 dilution of BD Brilliant Buffer for 30 min on ice. For analysis of mature myeloid cells, a staining cocktail containing Gr-1-Pacific Blue, Ly6C-BV605, Mac-1-PE/Cy7 and rat IgG in SM with a 1:4 dilution of Brilliant Buffer. Prior to analysis, BM cells were counterstained with 1 μg/mL propidium iodide (PI) and analyzed on a 3-laser, 12 channel FACSCelesta analyzer (Becton-Dickenson) or a 4-laser, 16 channel LSRII analyzer. For splenocyte analyses, spleens were minced through a 70 micron filter basket to create a single cell suspension, which was subsequently pelleted and depleted of red blood cells with 1x ACK as described above for BM cells. 1 × 10^7^ splenocytes were subsequently stained with an identical cocktail as above to read out mature hematopoietic cells.

For cell cycle analysis, 1 × 10^7^ RBC-depleted BM cells were stained with a variation of the BM HSPC cocktail, with each antibody stain performed on ice for 30 min: PE-Cy5-conjugated anti-CD3, CD4, CD5, CD8, Gr-1 and Ter119 as a lineage exclusion stain, Flk2-biotin, Sca-1-PE/Cy7, CD48-A700, and c-Kit-APC/Cy7, followed by a cocktail of 1:4 Brilliant Buffer:SM containing SA-BV605 and CD150-BV785. Cells were subsequently fixed and stained for Ki67 as described previously: After washing in SM, cells were fixed in Cytofix/Cytoperm buffer (Becton-Dickenson) for 20 min at room temperature (RT), washed in PermWash (BD) and permeablized using Perm Buffer Plus (BD) for 10 min at RT. Cells were again washed in PermWash, re-fixed in Cytofix/Cytoperm for 5 min, washed in PermWash and incubated with anti-Ki67-PerCP-Cy5.5 diluted in PermWash for 30 min at RT. Prior to analysis on an LSRII, cells were counterstained with DAPI diluted to 1 μg/mL in D-PBS.

For puromycin staining, an identical staining panel was used as for cell cycle analysis. Following fixation with Cytofix/Cytoperm performed as with cell cycle analysis, cells were stained with anti-puromycin antibody diluted in PermWash for 1 h at RT. Cells were subsequently washed in PermWash buffer, incubated with an anti-mouse IgG2a-APC secondary antibody for 30 min at RT, washed with PermWash and resuspended in SM for analysis on an LSRII.

For analysis of cells from liquid culture, cells were stained using a similar protocol as BM, except without RBC depletion. Cells were resuspended and half the content of the well was transferred to a FACS tube and stained with Sca-1-PE/Cy7, c-Kit-APC/Cy7, Mac-1-APC, FcγR-BV711, CD18-PE, MCSFR-BV605, and Gr1-Pacific Blue using an identical approach to that described for analysis of mature BM cells. Cells were analyzed on a 3-laser, 12-channel FACSCelesta (Becton-Dickenson). A complete list of antibodies including clone information, manufacturer and dilution can be found in Supplementary Table S3.

### Cell sorting

To analyze purified SLAM HSC and MPP^GM^, we harvested BM from all arm and leg bones as well as hips from mice by gently crushing bones in a mortar and pestle. BM cells were subsequently RBC depleted, passed through a 70 micron nylon mesh, and suspended on a Histopaque 1119 gradient (Sigma-Aldrich) to remove debris. BM was then enriched for c-Kit + cells by incubating on ice for 20 min with c-Kit microbeads, followed by column-based separation on an AutoMACS Pro magnetic cell separator using the Posseld2 setting. Enriched cells were subsequently stained as described above for BM HSPC analysis. Cells were subsequently sorted on a 4-laser FACSAria Fusion (Becton Dickenson) instrument equipped with a 100 micron nozzle.

### Cell culture

Purified cells were cultured using an identical protocol as previously published. Cells were grown for 4 days in culture-treated sterile 96-well plates containing StemPro 34 serum-free medium supplemented with L-Glutamine and Anti-anti (both 100x from Gibco), in addition to the following cytokines: SCF (25 ng/mL), TPO (25 ng/mL), IL-3 (10 ng/mL), GM-CSF (20 ng/mL), Flt3L (50 ng/mL), IL-11 (50 ng/mL), EPO (4 U/mL), and ±IL-1β (25 ng/mL) at 37°C in 5% CO_2_. For methylcellulose assays, 5 × 10^2^ cultured cells were transferred to 3 cm gridded dishes containing methylcellulose medium (StemCell Technologies; M3231) supplemented with the above cytokines and without IL-1β. Colonies were counted after 1 week and 1 × 10^4^ progeny cells were re-plated into fresh methylcellulose to measure serial clonogenic activity.

### Fluidigm qRT-PCR analysis

Cells were sorted from mouse bone marrow as described above and analyzed for gene expression as performed previously (Reynaud et al., 2011; Pietras et al., 2015; Pietras et al., 2016; Hernandez et al., 2020; Rabe et al., 2020; Chavez et al., 2021; Ahmed et al., 2022; Chavez et al., 2022). Cells were sorted at 100 cells per well in 5 μL CellsDirect reaction buffer (Invitrogen). RNA was then reverse transcribed and preamplified with a panel of 96 DeltaGene Assay primer sets (Fluidigm) for 20 rounds with Superscript III (Invitrogen) and subsequently treated with Exonuclease I (New England Biolabs) to remove non-target genetic material. cDNA was then diluted in DNA suspension buffer and loaded onto Fluidigm 96.96 Dynamic Array IFCs along with the DeltaGene Assay primers and run on a Biomark HD (Fluidigm) using SsoFast Sybr Green (Bio-Rad) as a detector. Data were analyzed using the ΔΔCT method and normalized to *Gusb* expression. Hierarchical clustering and PCA analyses were performed using ClustVis. As *Gusb* was used to normalize data, it was not included in clustering and PCA analysis. A complete list of all Fluidigm primer sequences can be found in Supplementary Table S4. *Hoxa2* and *Ebf1* were excluded from all analyses due to poor primer performance in our studies.

### ChIP-seq data mining

Primary ChIP-seq datasets from LSK/Flk2^-^/CD150^+^ cells and ChIP-seq data from PU-ER, BMDM and thioglycolate-elicited primary mouse macrophages GSE21512 were analyzed as previously published (Chavez et al., 2021). Fastq files were trimmed using Cutadapt and mapped to the mm10 mouse genome using HISAT2. Peak calling was performed using HOMER in factor mode with an FDR of <0.001. Intergenic peaks nearest to a TSS were annotated as the corresponding gene. Peak data were visualized from Bigwig files using the Internet Gene Viewer (IGV) application (igv.org/app).

### Statistical analysis

Statistical analyses were performed on GraphPad Prism v9.4.1. ANOVA with Tukey’s test were used for multivariate comparisons as described in the figure legends. *p*-values of 0.05 or less were considered statistically significant and are notated in the figures using asterisks.

## Results

### Chronic inflammation triggers aberrant myeloid expansion in PU.1-deficient mice

**FIGURE 1:**
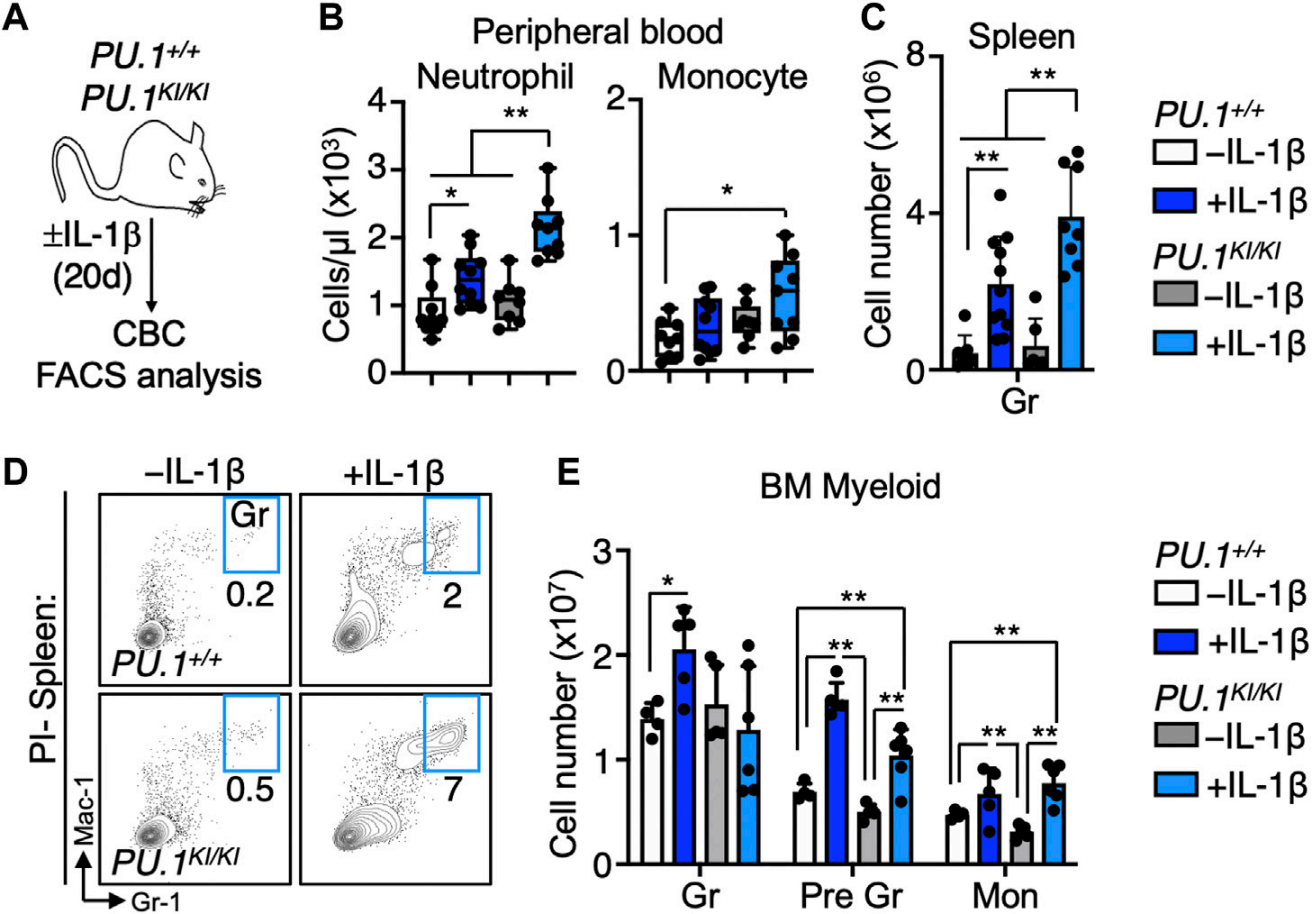
Chronic IL-1 triggers myeloid cell overproduction in PU.1-deficient mice. **(A)** Study design. *PU.1* ^*+/+*^ and *PU.1* ^*KI/KI*^ mice were treated for 20d ± IL-1β. **(B)** Complete blood count (CBC) analysis of myeloid cells in peripheral blood (*n* = 8–10/grp); box represents upper and lower quartiles with line representing median value. Whiskers represent minimum and maximum values. **(C)** Quantification of splenic granulocytes (Gr) and **(D)** representative FACS plots of splenic myeloid populations; individual values are shown with bars representing means. Error bars represent S.D. Data are compiled from two independent experiments. **(E)** Abundance of granulocytes, pre-granulocytes (Pre Gr) and monocytes (Mon) in the bone marrow (BM) (*n* = 4–6/grp). Individual values are shown with bars representing means. Error bars represent S.D. Data are compiled from two independent experiments. Statistical analysis for datasets in B-E was performed using ANOVA with Tukey’s test; *p < 0.05; **p < 0.01; ***p < 0.001.

To address the impact of PU.1 deficiency on the blood system under chronic inflammatory conditions, we analyzed the hematological parameters of *PU.1* ^*+/+*^ mice versus *PU.1* ^*KI/KI*^ mice (Staber et al., 2013) treated for 20 days ± IL-1β (0.5 μg/day via intraperitoneal injection) (Figure 1A). Complete blood counts (CBC) showed no abnormalities in the abundance of myeloid, lymphoid, and erythroid cells in PBS-treated control *PU.1* ^*KI/KI*^ mice relative to *PU.1* ^*+/+*^ controls (Figure 1B, Supplementary Figure S1A). Chronic IL-1β exposure triggered significant increases in peripheral blood neutrophils in *PU.1* ^*+ I +*^ mice, consistent with our prior published findings (Figure 1B). Strikingly, this phenotype was exacerbated in IL-1β-treated *PU.1* ^*KI/KI*^ mice. (Figure 1B). These alterations appeared confined to the myeloid lineage, as we observed no significant changes in lymphoid cell numbers and a similar degree of inflammation-induced anemia in these animals (Supplementary Figure S1A). Likewise, we observed aberrant myeloid expansion in the spleens of IL-1β-treated *PU.1* ^*KI/KI*^ mice relative to IL-1β-treated *PU.1* ^*+/+*^ mice, which was also accompanied by an overall increase in spleen mass (Figures 1C, D; Supplementary Figure S1B, C). In the bone marrow (BM), myeloid cell numbers in the BM of IL-1β-treated *PU.1* ^*KI/KI*^ mice were expanded with IL-1β treatment but not to an extent significantly different than their wild-type counterparts (Figure 1E), suggesting excess cells are likely being mobilized from the BM to the blood and spleen. Collectively, these data show that chronic inflammatory challenge elicits overproduction of myeloid cells in *PU.1* ^*KI/KI*^ mice.

**FIGURE 2:**
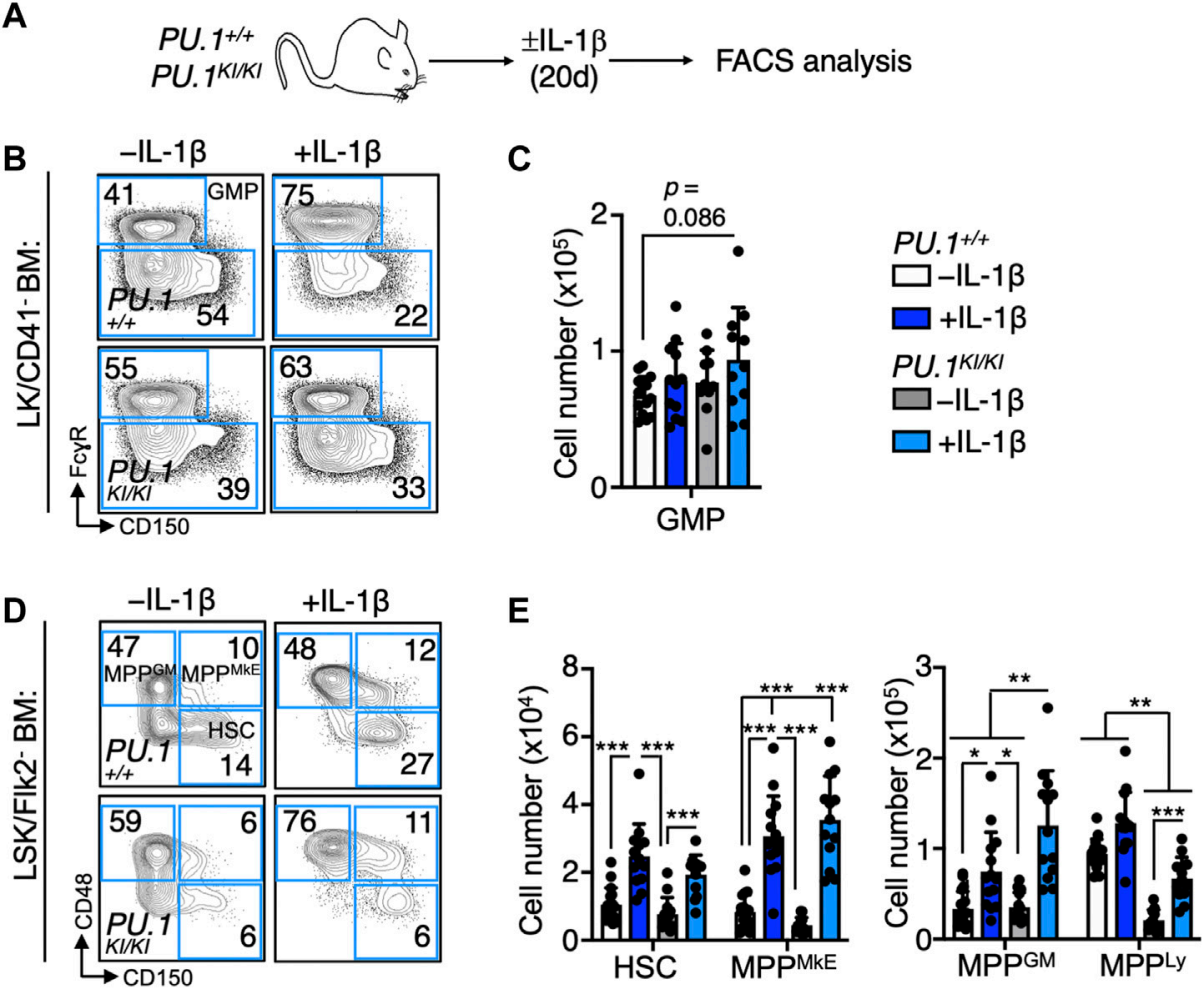
Chronic IL-1 induces aberrant expansion of PU.1-deficient MPP^GM^. **(A)** Study design. *PU.1* ^*+/+*^ and *PU.1* ^*KI/KI*^ mice were treated for 20d ± IL-1β. **(B)** Representative flow cytometry plots and **(C)** number of granulocyte macrophage progenitors (GMP) in the four long bones of mice (*n* = 10–14/grp); individual values are shown with bars representing means. Error bars represent S.D. Data are compiled from three independent experiments. **(D)** Representative flow cytometry plots and **(E)** number of defined HSPC populations in the four long bones of mice (*n* = 10–14/grp); individual values are shown with bars representing means. Error bars represent S.D. Data are compiled from three independent experiments. MPP^MkE^: MkE-primed MPP; MPP^GM^: GM-primed MPP; MPP^Ly^: Lymphoid-primed MPP. Statistical analysis for datasets in B-D was performed using ANOVA with Tukey’s test; *p < 0.05; **p < 0.01; ***p < 0.001.

To assess the impact of PU.1 deficiency on the dynamics of hematopoietic stem and progenitor (HSPC) populations in response to chronic IL-1β exposure, we analyzed the abundance of phenotypic HSPC in the BM of *PU.1* ^*KI/KI*^ and *PU.1* ^*+/+*^ mice treated for 20 days ± IL-1β (Figure 2A; Supplementary Figure S2A). We observed a trending increase in granulocyte/macrophage progenitors (GMP; Lin^−^/c-Kit^+^/CD41^-^/CD150^-^/FcγR^+^) (Pronk et al., 2007) in IL-1β-treated *PU.1* ^*KI/KI*^ BM relative to *PU.1* ^*+I+*^ controls. (Figures 2B, C). Chronic IL-1β also triggered significant expansion of HSC (HSC; Lin^−^/c-Kit^+^/Sca-1^+^/Flk2^-^/CD48^-^/CD150^+^) (Kiel et al., 2005) and MPP populations, specifically the megakaryocyte/erythroid-biased multipotent progenitor (MPP^MkE^; Lin^−^/c-Kit^+^/Sca-1^+^/Flk2^-^/CD48^+^/CD150^+^; also known as MPP2) and granulocyte/macrophage-biased (MPP^GM^; Lin^−^/c-Kit^+^/Sca-1^+^/Flk2^-^/CD48^-^/CD150^-^; also known as MPP3) in *PU.1* ^*+/+*^ mice (Figures 2D, E; Supplementary Figure S2A), consistent with prior reports (Cabezas-Wallscheid et al., 2014; Pietras et al., 2015; Challen et al., 2021). Notably however, expansion of MPP^GM^ was significantly potentiated in *PU.1* ^*KI/KI*^ mice relative to IL-1β-treated *PU.1* ^*+/+*^ controls (Figures 2D, E). Collectively, these data suggest the MPP^GM^ population may serve as a key axis of aberrant myeloid expansion following chronic inflammatory challenge in *PU.1* ^*KI/KI*^ mice.

### IL-1β triggers aberrant cell cycle activity PU.1-deficient MPP^GM^

**FIGURE 3:**
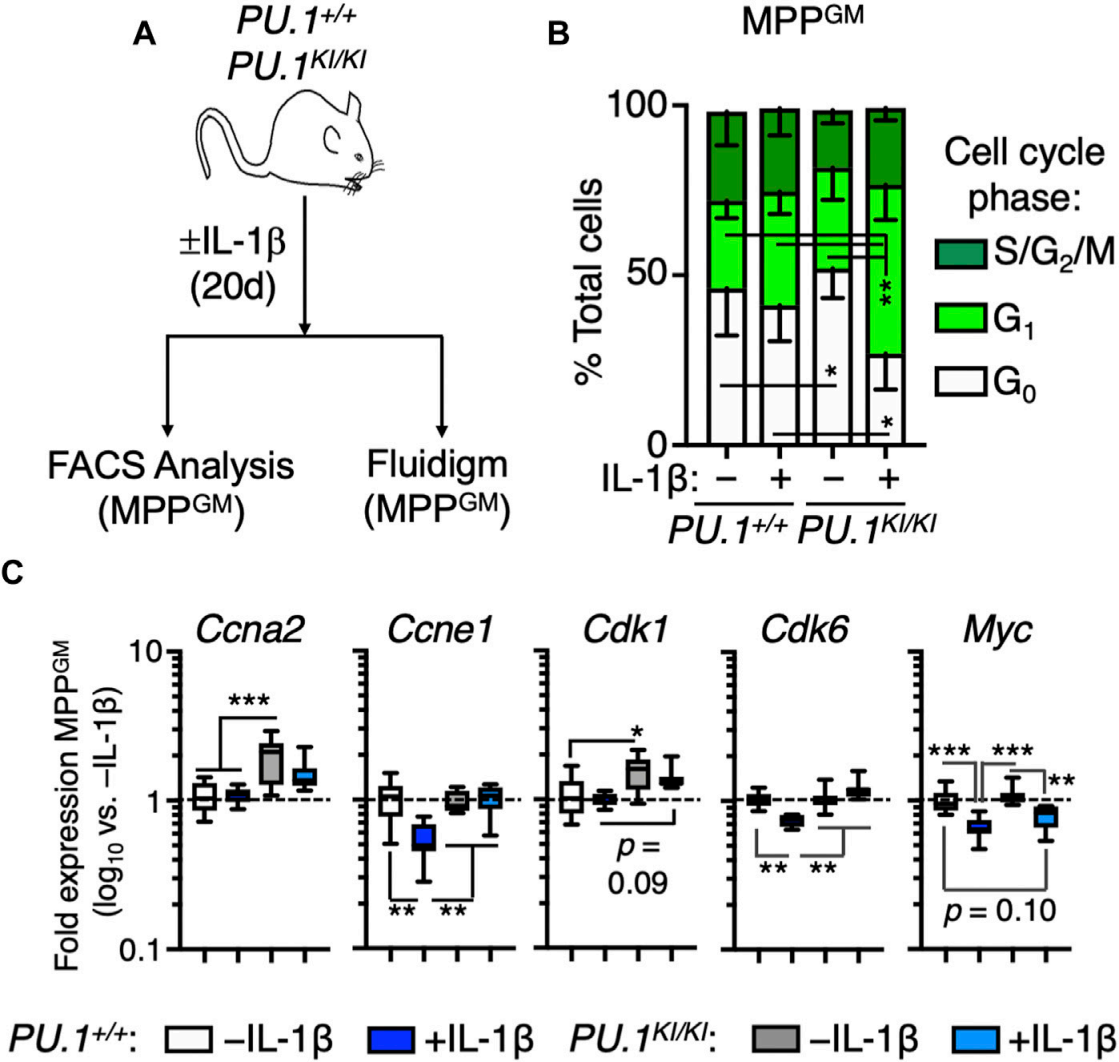
Chronic IL-1 triggers aberrant cell cycle activity in PU.1-deficient MPP^GM^. **(A)** experiments. *PU.1* ^*+/+*^ and *PU.1* ^*KI/KI*^ mice were treated for 20d ± IL-1β. **(B)** Quantification of cell cycle distribution in MPP^GM^ (n = 4–5/grp). Stacked bars show means for each cell cycle phase measured. Error bars represent S.D. Data are compiled from two independent experiments. **(C)** Cell cycle gene expression in MPP^GM^ (*n* = 8/group). Data are expressed as log_10_ fold expression versus -IL-1β. Box represents upper and lower quartiles with line representing median value. Whiskers represent minimum and maximum values. Data represent two independent experiments. Statistical analysis for datasets in B-C was performed using ANOVA with Tukey’s test; *p < 0.05; **p < 0.01; ***p < 0.001.

We previously showed that PU.1 deficiency leads to increased proliferation in HSC^LT^ following IL-1β stimulation, thereby driving expansion of these cells *in vivo* (Chavez et al., 2021). To assess whether MPP^GM^ expansion was likewise related to increased cell cycle activity, we analyzed cell cycle distribution via Ki67/DAPI staining in *PU.1* ^*KI/KI*^ MPP^GM^ following treatment for 20 days ± IL-1β (Figure 3A). While MPP^GM^ from *PU.1* ^*KI/KI*^ mice −IL-1β exhibited a higher proportion of cells in G_0_ relative to *PU.1* ^*+/+*^ controls, IL-1β treatment significantly and selectively potentiated cell cycle activity in *PU.1* ^*KI/KI*^ MPP^GM^ mice (Figure 3B; Supplementary Figure S2B). Taken together, these data indicate that IL-1β triggers increased cell cycle activity in *PU.1* ^*KI/KI*^ MPP^GM^, thereby contributing to the aberrant expansion of this population.

Next, we analyzed gene expression patterns in *PU.1* ^*+/+*^ and *PU.1* ^*KI/KI*^ MPP^GM^ from mice treated for 20 days ± IL-1β using our custom Fluidigm qRT-PCR gene expression array. This approach allowed us to measure expression of 94 genes critical for HSPC function (Supplementary Table S4). As anticipated, expression of *Spi1* (*PU.1*) was significantly reduced in *PU.1* ^*KI/KI*^ MPP^GM^ (Supplementary Figure S3A). IL-1β was still able to induce *Spi1* expression in *PU.1* ^*KI/KI*^ MPP^GM^, albeit at significantly reduced levels relative to *PU.1* ^*+/+*^ MPP^GM^ (Supplementary Figure S3A), likely reflecting the capacity of inflammation-induced signals to trigger PU.1 expression independently of the −14 kb URE PU.1 autoregulatory binding site (Ahmed et al., 2022). Furthermore, we did not notice overt defects in *Il1r1* expression levels in *PU.1* ^*KI/KI*^ MPP^GM^ (Supplementary Figure S3A). To evaluate overall differences in gene expression between *PU.1* ^*+/+*^ and *PU.1* ^*KI/KI*^ MPP^GM^ ± IL-1β, we next performed hierarchical clustering analysis (Pearson correlation with average linkage). MPP^GM^ samples clustered predominantly by genotype and secondarily by treatment condition (Supplementary Figure S3B). MPP^GM^ samples likewise were clearly distinguished by principal component analysis (PCA), with PC1 appearing to discriminate samples based on relative PU.1 activity and PC2 distinguishing *PU.1* ^*+/+*^ controls from all other samples (Supplementary Figure S3C), collectively indicating unique gene programs present in each genotype and treatment. Given the changes in cell cycle activity triggered by IL-1β, we examined the expression of cell cycle genes in MPP^GM^. Notably, genes such as *Ccne1*, *Cdk6 and Myc* were repressed in *PU.1* ^*+/+*^ MPP^GM^ following IL-1β exposure (Figure 3C). However *PU.1* ^*KI/KI*^ MPP^GM^ failed to robustly repress these genes following *in vivo* IL-1β treatment (Figure 3C). Collectively, these data support a model in which PU.1 is required to repress expression of cell cycle genes to maintain normal MPP^GM^ cell cycle activity following chronic inflammatory challenge.

### PU.1-deficient MPP^GM^ retain self-renewal gene programs following IL-1β exposure

**FIGURE 4:**
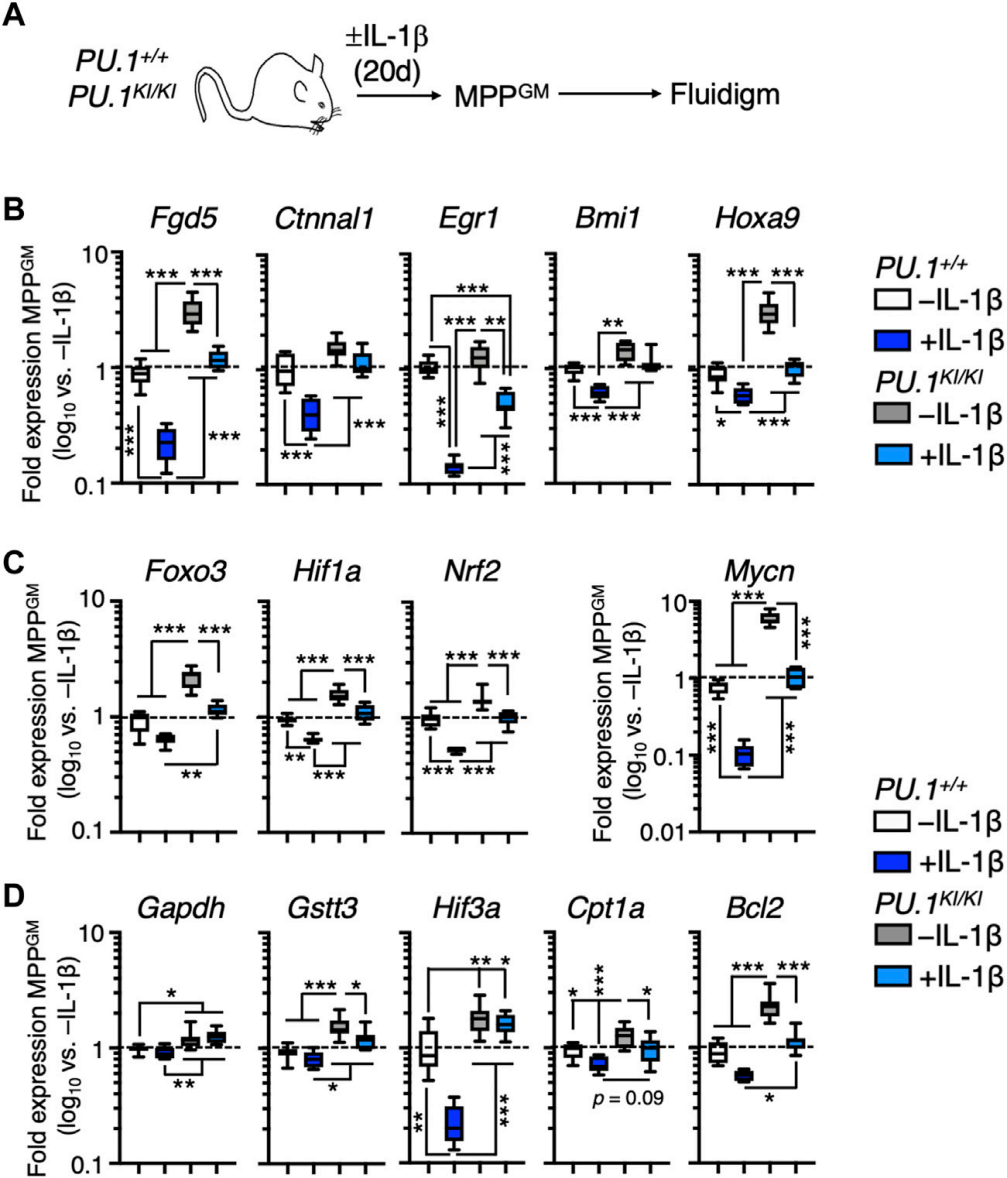
PU.1-deficient MPP^GM^ retain expression of self-renewal genes under inflammatory stress. **(A)** Study design for Fluidigm qRT-PCR array studies. *PU.1* ^*+/+*^ and *PU.1* ^*KI/KI*^ mice were treated for 20d ± IL-1β. **(B)** Quantification of genes associated with HSC function in MPP^GM^ (*n* = 8/group). Quantification of **(C)** self-renewal-associated transcription factors and **(D)** target genes in MPP^GM^ (*n* = 8/group). Data are expressed as log_10_ fold expression versus -IL-1β. Box represents upper and lower quartiles with line representing median value. Whiskers represent minimum and maximum values. Data are representative of two independent experiments. Statistical analysis for datasets in B-D was performed using ANOVA with Tukey’s test; *p < 0.05; **p < 0.01; ***p < 0.001.

While PU.1-deficient MPP^GM^ exhibit increased cell cycle activity, concomitant disruption of gene programs associated with differentiation is likely required for their selective expansion under chronic inflammatory challenge. Thus, we surveyed expression of key genes associated with self-renewal in MPP^GM^ from mice treated for 20 days ± IL-1β (Figure 4A). Consistent with the capacity of IL-1β to trigger rapid myeloid differentiation in HSPC, expression of these genes was significantly reduced in *PU.1* ^*+/+*^ MPP^GM^ following IL-1β exposure (Figure 4B). Strikingly, relative to *PU.1* ^*+/+*^ MPP^GM^, *PU.1* ^*KI/KI*^ MPP^GM^ exhibited aberrantly high baseline expression of *Fgd5*, *Ctnnal1*, *Egr1*, *Bmi1* and *Hoxa9* (Figure 4B). These genes were nonetheless downregulated following IL-1β exposure, but only to levels found at steady state in *PU.1* ^*+/+*^ MPP^GM^ (Figure 4B). These data suggest IL-1β-mediated repression of these genes may involve other transcriptional regulators aside from PU.1 including CEBPA, which regulates many of the same genes (Koschmieder et al., 2005; Pundhir et al., 2018; Higa et al., 2021). Along these lines, we observed a significant increase in *Cebpa* expression in *PU.1* ^*KI/KI*^ MPP^GM^ relative to *PU.1* ^*+/+*^ MPP^GM^ following IL-1β treatment (Supplementary Figure S3D), suggesting *Cebpa* expression may be induced to compensate for PU.1 loss in *PU.1* ^*KI/KI*^ MPP^GM^ during chronic inflammatory challenge.

To further examine the impact of PU.1 deficiency on MPP^GM^, we examined the expression of key genes regulating mechanisms that maintain stem cell activity in the hematopoietic system, specifically forkhead box O3 (*Foxo3*), hypoxia-inducible factor-1α (*Hif1a*), nuclear regulatory factor-2 (*Nrf2*), and N-Myc (*Mycn*) (Figure 4C). These transcription factors regulate numerous gene programs required for stem cell function such as autophagy, glycolytic metabolism, the antioxidant response, and apoptosis resistance. IL-1β exposure triggered robust repression of these genes in *PU.1* ^*+/+*^ MPP^GM^. Interestingly, *PU.1* ^*KI/KI*^ MPP^GM^ again exhibited increased baseline expression of these factors (Figure 4C) While IL-1β exposure likewise downregulated their expression in *PU.1* ^*KI/KI*^ MPP^GM^, expression was again only reduced to levels found in *PU.1* ^*+/+*^ MPP^GM^ at steady state (Figure 4C). Furthermore, we observed similar expression patterns in key downstream target genes of these transcription factors, including genes regulating glycolysis (glyceraldehyde-3 phosphate dehydrogenase; *Gapdh*), antioxidant activity (glutathione S-transferase T3; *Gstt3*), hypoxia response (hypoxia-inducible factor 3a; *Hif3a*), fatty acid oxidation (carnitine palmitoyltransferase 1a; *Cpt1a*), and survival (B-cell leukemia 2; *Bcl2*) (Figure 4D). Taken together, our data suggest PU.1-deficient HSPC may be metabolically poised to support cell cycle activity, while maintaining self-renewal activity via disruption of differentiation gene programs. These observations are broadly consistent with prior work showing that PU.1 binds to and represses key genes in glycolysis and lipid biosynthesis pathways used for production of energy and anabolic factors that support cell proliferation (Solomon et al., 2017).

We next assessed whether the genes investigated above possess PU.1 binding sites. Thus, we queried our previously published PU.1 ChIP-seq datasets in which we assessed PU.1 binding in LSK/Flk2^−^/CD150^+^ HSPC. We also compared these results with data from three other publicly available PU.1 ChIP-seq datasets (Heinz et al., 2010) in bone marrow-derived macrophages (BMDM), thioglycolate-elicited macrophages, and the PU-ER cell line (Walsh et al., 2002), which is derived from PU.1-deficient fetal HSPC expressing a tamoxifen-inducible *PU.1* transgene. Notably, our ChiP-seq dataset identified PU.1 peaks associated with a majority of the genes identified in Figure 4 in primary HSPC (Supplementary Figure S4A–D; Supplementary Table S1). Furthermore, these PU.1 peaks were present in at least two of the other publicly available datasets. Collectively, these data support a model in which PU.1 negatively regulates expression of numerous self-renewal genes, with PU.1-deficient MPP^GM^ consequently retaining high expression levels of these factors. These data are strongly reminiscent of *Cebpa*-deficient MPP^GM^, which likewise retained high expression levels of stem cell genes including *Foxo3*, *Mycn*, *Bmi1* and *Bcl2* following chronic exposure to IL-1β (Higa et al., 2021).

### PU.1-deficient MPP^GM^ exhibit impaired differentiation in response to IL-1β

**FIGURE 5:**
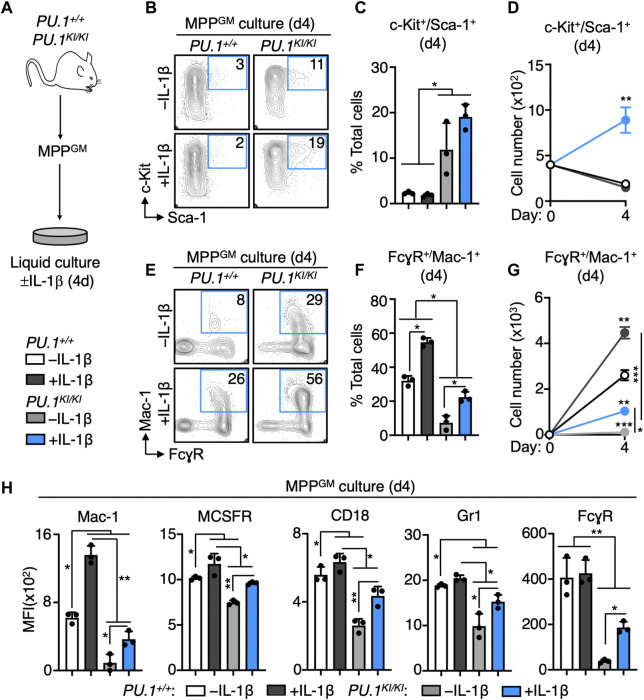
PU.1-deficient MPP^GM^ exhibit aberrant expansion and impaired myeloid differentiation during IL-1 stimulation *in vitro*. **(A)** Study design for culture experiments. FACS-purified MPP^GM^ were cultured in serum-free medium for 4 days ± IL-1β in myeloid growth conditions (*n* = 3/grp). **(B)** Representative FACS plots, **(C)** frequency and **(D)** number of phenotypically immature (c-Kit^+^/Sca-1^+^) cells after 4d culture. **(E)** Representative FACS plots, **(F)** frequency and **(G)** number of phenotypically myeloid-committed (FcγR^+^/Mac-1^+^) cells after 4d culture. **(H)** Surface expression of myeloid lineage markers. Data are expressed as mean fluorescence intensity (MFI). For bar graphs, individual values are shown with bars representing means. For line graphs, mean values are shown. Error bars represent S.D. Data are representative of two individual experiments. Statistical analysis for datasets in C-H was performed using ANOVA with Tukey’s test; *p < 0.05; **p < 0.01; ***p < 0.001.

Given our gene expression data, we hypothesized that *PU.1* ^*KI/KI*^ MPP^GM^ would exhibit impaired capacity to differentiate in response to IL-1β. As *PU.1* ^*KI/KI*^ HSPC fail to engraft in transplantation assays, and to minimize potential impacts of BM niche signals altered by IL-1β *in vivo*, we used a well-defined *in vitro* liquid culture system to study the impact of PU.1 deficiency on the differentiation kinetics of purified MPP^GM^ in response to *in vitro* IL-1β stimulation (Figure 5A) (Pietras et al., 2016; Chavez et al., 2021; Higa et al., 2021). After 4 days of culture, we analyzed the frequency and number of immature (c-Kit^+^/Sca-1^+^) and myeloid-committed (FcγR^+^/Mac-1^+^) cells in the cultures. Strikingly, we observed a significant increase in the proportion of c-Kit^+^/Sca-1^+^ immature cells in *PU.1* ^*KI/KI*^ MPP^GM^ cultures relative to *PU.1* ^*+/+*^ control cells regardless of IL-1β stimulation (Figures 5B–D). We subsequently confirmed the increased proportion of immature cells in the day 4 *PU.1* ^*KI/KI*^ MPP^GM^ cultures via serial clonogenic assay (Supplementary Figure S4A, B). Conversely, we observed a lower proportion and number of myeloid-committed *PU.1* ^*KI/KI*^ MPP^GM^ during the culture period (Figures 5E–G), including a lower overall proportion of myeloid-committed *PU.1* ^*KI/KI*^ MPP^GM^ in the -IL-1β cultures, in line with our gene expression and c-Kit^+^/Sca-1^+^ culture data. To better understand the impact of PU.1 deficiency on myeloid surface marker expression, we analyzed surface expression of a broader panel of myeloid markers in our cultures, including CD18, MCSFR and Gr-1. Unstimulated *PU.1* ^*KI/KI*^ MPP^GM^ exhibited significantly lower surface expression of each of these markers after 4 days culture (Figure 5H). In line with these findings, we identified multiple PU.1 peaks associated with MCSFR (*Csfr1*), FcγR (*Fcgr2b*), CD18 (*Itgb2*), Gr-1 (*Ly6c/Ly6g*) and Mac-1 (*Itgam*, as previously reported in (Chavez et al., 2021)) in our ChIP-seq datasets, consistent with the well-known role of PU.1 in directly binding and transducing these genes (Supplementary Figure S5C; Supplementary Table S2) (DeKoter et al., 1998; Singh et al., 1999; DeKoter and Singh, 2000; DeKoter et al., 2007). IL-1β nonetheless accelerated expression of all five myeloid surface markers in *PU.1* ^*KI/KI*^ MPP^GM^, again to levels roughly equivalent to unstimulated WT cells (Figure 5H). Hence, reduced PU.1 expression in *PU.1* ^*KI/KI*^ MPP^GM^ delays the activation of myeloid differentiation programs, leading to retention of an immature phenotype following IL-1β stimulation.

## Discussion

PU.1 is a well-known master regulator of hematopoietic stem cell function and lineage determination (Koschmieder et al., 2005). Here, we use the *PU.1* ^*KI/KI*^ mouse model of PU.1 deficiency to address the role of PU.1 in regulating myelopoietic activity following chronic inflammatory challenge with IL-1β. We find that PU.1 deficiency leads to overproduction of mature myeloid cells and aberrant expansion of MPP^GM^, a progenitor population that serves as an “emergency” reservoir for myeloid cell production (Pietras et al., 2015), following chronic IL-1β treatment. Further, we show that *PU.1* ^*KI/KI*^ MPP^GM^ exhibit aberrant cell cycle activity, retain high levels of self-renewal gene expression and exhibit delayed myeloid differentiation in response to IL-1β signaling. Altogether, our data show that PU.1 plays a critical role in regulating inflammation driven HSPC expansion and myelopoiesis by ensuring appropriate regulation of self-renewal and differentiation genes in addition to restraining cell cycle activity.

“Emergency” myelopoiesis is a critical response to physiological insults that supplies the host with enough innate immune cells to fight infections and/or contribute to the repair and immunosurveillance of damaged tissues (Caiado et al., 2021; Collins et al., 2021). The mechanisms regulating “emergency” myelopoiesis are likely multifactorial, and transcriptional regulators such as C/EBPβ have also been implicated as drivers of hematopoietic responses to injury and infection (Hirai et al., 2006; Hirai et al., 2015). We and others have shown that PU.1 plays a key role in this process (Pietras et al., 2016; Yamashita and Passegue, 2019; Hernandez et al., 2020; Rabe et al., 2020; Chavez et al., 2021; Ahmed et al., 2022; Chavez et al., 2022). We found that IL-1β rapidly and robustly induces PU.1 expression in HSC and MPP populations (Pietras et al., 2016), including MPP^GM^. Increased PU.1 expression in turn triggers enhanced activation of myeloid differentiation pathways in HSPC, leading to increased myeloid cell output, sometimes referred to as “myeloid-biased” hematopoiesis (Pietras et al., 2016; Rabe et al., 2020; Ahmed et al., 2022; Chavez et al., 2022). We recently showed that PU.1 also functions to prevent spurious HSC proliferation and expansion of the HSC pool during chronic inflammatory stress by repressing induction of cell proliferation gene programs during chronic IL-1β treatment (Chavez et al., 2021). With this body of work in mind, our study was motivated by the hypothesis that PU.1 may play a critical role in limiting myeloid output in response to inflammatory stress. Here, our data show that *PU.1* ^*KI/KI*^ mice produce an overabundance of neutrophils and monocytes in response to chronic IL-1β treatment, facilitated by aberrant proliferation and expansion of myeloid-biased MPP^GM^. We also find that *PU.1* ^*KI/KI*^ MPPGM exhibit impaired differentiation in response to IL-1β, characterized by high levels of self-renewal gene expression and delayed expression of key myeloid surface markers. Hence, while PU.1 serves to redirect HSC fate toward the myeloid lineage, it also limits the magnitude of the hematopoietic response via restricting the size of HSC and MPP pools that give rise to myeloid cell populations. Our data thus support a model in which PU.1 serves dual, and complementary roles: 1) facilitating proper myeloid differentiation and 2) constraining myelopoietic responses to physiological insults.

Our data show that PU.1 is required to repress cell cycle genes and cell cycle activity in MPP^GM^ following IL-1β exposure. Previous work has shown that PU.1 restricts the cell cycle activity of myeloid-committed progenitors (Kueh et al., 2013), thereby facilitating homeostatic myeloid differentiation via accumulation of sufficient myeloid lineage determinants prior to cell division. PU.1 similarly represses cell cycle genes in T cell progenitors, and thus cell cycle restriction may play a similar role in lymphoid development (Kueh et al., 2013; Staber et al., 2013; Champhekar et al., 2015). We previously showed that PU.1 likewise rapidly represses cell cycle genes in HSC following inflammatory insults, and thus limits ongoing proliferation and expansion of the phenotypic HSC pool in response to chronic inflammatory stimulation (Chavez et al., 2021). Hence, PU.1 functions as a “braking” mechanism that rheostatically suppresses proliferative activity in multiple HSPC populations during an inflammatory insult, allowing for sufficient myeloid expansion for host defense while preventing aberrant expansion of progenitor pools (Caiado et al., 2021). Notably, we find that like *PU.1* ^*KI/KI*^ HSC (Chavez et al., 2021), *PU.1* ^*KI/KI*^ MPP^GM^ constitutively overexpress cell cycle genes under homeostatic conditions, but do not exhibit increases in cell cycle activity without inflammatory stimulation. These data indicate that overexpression of cell cycle genes establishes a nascent phenotype that is not sufficient to drive aberrant cell cycle activity absent an inflammatory trigger. Indeed, we and others have shown that IL-1β promotes hematopoietic regeneration and is capable of briefly driving HSPC into the cell cycle, in large part via activation of the PI3K-AKT pathway (Hemmati et al., 2019). While these findings (overexpression of cell cycle genes without increased cell cycle activity) may at first appear paradoxical, it is well known that the activation of mitogenic pathways such as PI3K-AKT by inflammatory signals induces the necessary post-translational modifications (e.g., phosphorylation) of cell cycle regulatory proteins to potentiate cell cycle progression (Chang et al., 2003; Pietras et al., 2011; Warr et al., 2011; Pietras et al., 2014). As PI3K-AKT signaling can also modulate the activity of PU.1 via phosphorylating it (Rieske and Pongubala, 2001), further studies can address the extent to which the PI3K-AKT pathway drives IL-1β-dependent gene regulation by PU.1.

We find that *PU.1* ^*KI/KI*^ MPP^GM^ constitutively overexpress key genes associated with self-renewal. These data align with our analyses of PU.1 ChIP-seq datasets, which identified PU.1 binding sites at most of these targets. These data indicate PU.1 may directly repress their expression. Our previously published analysis (Higa et al., 2021) of *Cebpa*-deficient MPP^GM^ shows nearly identical patterns of overexpression in genes like *Bmi1*, *Foxo3* and *Mycn* (Tothova et al., 2007; Takubo et al., 2010; Merchant et al., 2011; Warr et al., 2013; Murakami et al., 2014; Scognamiglio et al., 2016). These data are consistent with the roles of PU.1 and CEBPA as joint regulators of myeloid differentiation. Despite reduced PU.1 expression levels, IL-1β treatment still triggered repression of self-renewal genes in *PU.1* ^*KI/KI*^ MPP^GM^, though expression levels of these genes remained higher than their WT counterparts due to elevated homeostatic expression levels. It is noteworthy that we observed identical patterns of gene expression (Higa et al., 2021) in *Cebpa*-deficient MPP^GM^. Along these lines, we previously found that both CEBPA and PU.1 bind genes induced or repressed by IL-1β in MPP^GM^, including stem cell genes and myeloid lineage determinants (Higa et al., 2021). Hence, CEBPA is likely able to partially compensate for loss of PU.1 in driving myeloid differentiation, and indeed here we find *Cebpa* expression is significantly increased in *PU.1* ^*KI/KI*^ MPP^GM^ by chronic IL-1β exposure. Hence, our data support a model in which PU.1 and CEBPA are critical for establishing the homeostatic ‘set point’ for numerous self-renewal and myeloid differentiation gene programs and jointly contribute to their regulation in response to IL-1β. Of note, we also found that expression of *PU.1/Spi1* itself was upregulated by IL-1β in *PU.1* ^*KI/KI*^ MPP^GM^. As *PU.1* ^*KI/KI*^ mice lack the PU.1 autoregulatory binding site at the −14 kb enhancer, the mechanism of IL-1β-driven upregulation of *Spi1* may be driven by pathways such as NF-κB (Pietras et al., 2016; Etzrodt et al., 2019; Yamashita and Passegue, 2019; Ahmed et al., 2022), which we and others previously showed to be crucial for PU.1 induction in HSC. Increased PU.1 expression in may also occur in *PU.1* ^*KI/KI*^ MPP^GM^ via CEBPA-mediated transduction, as CEBPA binds to and induces *Spi1* expression in HSPC (Kummalue and Friedman, 2003; Yeamans et al., 2007). Further studies can directly address the dynamics of PU.1, NF-κB and CEBPA in response to inflammatory cues in HSC and their downstream progenitors. Moreover, several other transcriptional regulators interact with PU.1, including AP-1 family transcription factors (Steidl et al., 2006; Boasman et al., 2019; Zhao et al., 2022). Indeed, the AP-1 factors c-Jun and JunB are also critical interacting partners with PU.1 that also regulate myeloid differentiation (including PU.1 expression) (Steidl et al., 2006; Raghav et al., 2018; Zhao et al., 2022). PU.1 can also engage in competitive interactions with transcriptional regulators such as GATA1 to promote myeloid differentiation (Strasser et al., 2018; Wheat et al., 2020; Raghav and Gangenahalli, 2021). Thus, reduced PU.1 expression in *PU.1* ^*KI/KI*^ HSPC likely initiates a ‘ripple effect’ that disrupts additional transcriptional networks controlling the balance between myeloid differentiation and self-renewal.

PU.1 also plays important roles in establishing the functional properties of mature myeloid cells (Singh et al., 1999). PU.1 regulates numerous gene programs associated with host defense and immune function, including expression of MHC and costimulatory genes, as well as immune checkpoints and immune effector genes (Fisher et al., 1998; Karpurapu et al., 2011; Kitamura et al., 2012; Keightley et al., 2017). Further work should address the extent to which myeloid cells overproduced in response to inflammation in the *PU.1* ^*KI/KI*^ mouse model are functionally mature and/or have the potential contribute to tissue dysfunction in the setting of chronic disease. Of note, pathogenic myeloid cell activity in the context of autoimmunity and chronic inflammatory disease has been attributed to PU.1-dependent gene programs (Fang et al., 2022), raising the question as to whether PU.1 activity constitutes a potential therapeutic target. Given the association between impaired PU.1 network function and leukemogenesis, directly targeting PU.1 activity in a specific manner without compromising normal hematopoietic or immune function could be highly challenging. Targeting the pathogenic inflammatory processes that potentiate PU.1 activity and contribute to other pathogenic disease features may instead be optimal. Therapeutic modalities targeting pathogenic cytokines such as IL-1 and TNF are already in widespread clinical use (Davignon et al., 2013). Along these lines, we recently showed that IL-1R blockade could reduce the expression of PU.1 target genes in HSC closer to homeostatic levels, with concurrent reductions in myeloid output (Hernandez et al., 2020). Further studies can address the extent to which inflammation blockade normalizes PU.1 activity in mature and immature hematopoietic cells.

Dysregulation of the PU.1 transcriptional network is a common phenotype associated with hematological malignancy, particularly diseases of the myeloid lineage (Cook et al., 2004; Dakic et al., 2005; Koschmieder et al., 2005; Will et al., 2015; Sive et al., 2016; Aivalioti et al., 2022). We had previously shown that loss of PU.1 in conjunction with IL-1β signaling could trigger aberrant expansion of HSC. Here, we show that under chronic inflammatory conditions, reduced PU.1 expression is sufficient to induce a myeloproliferative phenotype characterized by aberrant accumulation of mature myeloid cells in the blood and spleen. These findings are consistent with prior work indicating PU.1 can constrain myelopoietic activity (DeKoter et al., 1998; Dakic et al., 2005), with our data extending these findings to inflammatory conditions. In addition to mature myeloid progeny, we observe expansion of HSPC, specifically the ‘myeloid-biased’ MPP^GM^. It is noteworthy that MPP^GM^ (also commonly referred to as MPP3) (Challen et al., 2021) appears to be a nexus of myeloid expansion under both inflammatory conditions and in animal models of myeloid malignancy (Schepers et al., 2013; Pietras et al., 2015; Shih et al., 2015; Pietras et al., 2016; Herault et al., 2017; Hernandez et al., 2020; Kang et al., 2020; Rabe et al., 2020). As MPP^GM^ has been considered a component of the “emergency” hematopoietic response, these data support a model in which the MPP^GM^ differentiation pathway is essentially hijacked by oncogenic mutations, serving as an engine of abnormal myeloid expansion. In this setting, oncogenic mutations in signaling pathway genes such as Ras and Flt3 that trigger downstream mitogenic activity could serve a similar function as inflammatory signaling in our model, triggering abnormal expansion of HSPC. Of note, recent work has shown that loss-of-function mutations in *TET2*, leads to hypermethylation at PU.1 binding sites throughout the genome, disrupting the PU.1 transcriptional network (Aivalioti et al., 2022). Indeed, *Tet2*-deficient HSPC exhibit similar characteristics to *PU.1*-deficient HSPC, namely the capacity to undergo accelerated expansion in the context of chronic inflammation, coupled with altered cell cycle activity (Consortium et al., 2007; Caiado et al., 2022). These data point to PU.1 disruption as a likely mechanism driving inflammatory expansion of mutant HSPC during leukemic evolution.

Taken together, our data show that PU.1 expression is required to restrain production of myeloid cells and their hematopoietic precursors in response to chronic inflammation. Hence, loss of PU.1 expression is sufficient to support aberrant myelopoiesis and HSPC expansion in this setting. Thus, our data support a model in which loss of PU.1 function broadly impacts the size and function of the myeloid hematopoietic hierarchy in addition to driving abnormal expansion of the long-term HSC pool. Further studies should address the mechanism(s) by which dysregulation of self-renewal gene programs, particularly those regulating cellular metabolism, drive aberrant MPP^GM^ expansion and inflammatory myelopoiesis in PU.1-deficient settings. Disrupting the PU.1 network could thus serve as a common mechanistic driver by which leukemia-associated mutations initiate selective expansion of mutant cells in the setting of aging- and disease-related chronic inflammation.

## Data Availability

The datasets presented in this study can be found in online repositories. The names of the repository/repositories and accession number(s) can be found in the article/Supplementary Material.

## Supplementary material

SUPPLEMENTARY FIGURE S1 Analysis of hematopoietic populations in PU.1-deficient mice. **(A)** CBC analysis of lymphoid and red blood cell (RBC) populations in peripheral blood of mice in Figure 1B (*n* = 8–10/grp); box represents upper and lower quartiles with line representing median value. Whiskers represent minimum and maximum values. Data are compiled from two independent experiments. **(B)** Spleen masses from mice in Figure 1C (*n* = 4/grp); individual values are shown with bars representing means. For line graphs, mean values are shown. Error bars represent S.D. Data are from one experiment. **(C)** Representative flow cytometry plots showing gating strategy for identification of myeloid cell populations in the BM of mice in Figure 1E. Statistical analysis for datasets in A-B was performed using ANOVA with Tukey’s test; **p* < 0.05; ***p* < 0.01; ****p* < 0.001.

SUPPLEMENTARY FIGURE S2 Gating strategies for analysis of HSPC populations. **(A)** Representative flow cytometry plots and gating strategy for identification of HSPC populations from the BM of mice in Figure 2E. **(B)** Representative flow cytometry plots showing cell cycle distribution in MPP^GM^ from mice in Figure 3B. Cell cycle phases (G_0_, G_1_, S/G_2_/M) are defined by Ki67 and DAPI staining as shown.

SUPPLEMENTARY FIGURE S3 Fluidigm gene expression analysis of MPP^GM^. **(A)** *PU.1* ^*+/+*^ and *PU.1* ^*KI/KI*^ mice were treated for 20d ± IL-1β. Expression levels of PU.1 (*Spi1*), Mac-1 (*Itgam*) and IL-1 receptor (*Il1r1*) in MPP^GM^ (*n* = 8/group). Data are expressed as log_10_ fold expression versus -IL-1β. Box represents upper and lower quartiles with line representing median value. Whiskers represent minimum and maximum values. Data are representative of two individual experiments. **(B)** Hierarchical clustering of MPP^GM^ Fluidigm array data (Pearson correlation with average linkage) and **(C)** principal component analysis (PCA; top) and PCA loading plot (bottom). Analyses were performed using ClustVis. **(D)** Expression levels of *Cebpa* in MPP^GM^ Box represents upper and lower quartiles with line representing median value. Whiskers represent minimum and maximum values. Data are representative of two individual experiments. Statistical analysis for data in A and D was performed using ANOVA with Tukey’s test; **p* < 0.05; ***p* < 0.01; ****p* < 0.001.

SUPPLEMENTARY FIGURE S4 PU.1 ChIP-seq peak analysis of cell cycle and self-renewal genes. Visualization of PU.1 peaks in representative cell cycle versus whole-cell extract controls. **(A)**, HSC marker **(B)**, Self-renewal transcription factor **(C)** and target **(D)** genes identified in Figures 3, 4 from ChIP-seq analysis of wild-type HSPC. For each gene, chromosomal location and gene maps are shown, as are peak height scales. WCE: whole cell extract. Red boxes identify PU.1 peaks within the viewing window that correspond to data shown in Supplementary Table S1.

SUPPLEMENTARY FIGURE S5 Clonogenic potential in PU.1-deficient MPP^GM^ and PU.1 ChIP-seq analysis of myeloid marker genes. **(A)** Study design and **(B)** analysis of serial clonogenic activity in MPP^GM^ liquid cultures after 4 days ± IL-1β. Individual values are shown with bars representing means. Data are from one experiment. **(C)** Visualization of PU.1 peaks from ChIP-seq analysis of wild-type HSPC. For each gene, chromosomal location and gene maps are shown, as are peak height scales. WCE: whole cell extract. Red boxes identify PU.1 peaks within the viewing window that correspond to data shown in Supplementary Table S2. Statistical analysis for data in B was performed using ANOVA with Tukey’s test **p* < 0.05; ***p* < 0.01; ****p* < 0.001.

SUPPLEMENTARY TABLE S1 ChIP-seq peak locations in cell cycle and self-renewal genes. PU.1 ChIP-seq peak locations, scores and PU.1 motif scores. Data also show peak overlaps with publicly available datasets (0 = no peak present; 1 = peak present). Only PU.1 ChIP-seq peaks overlapping with peaks in ≥2 publicly available datasets were considered.

SUPPLEMENTARY TABLE S2 ChIP-seq peak locations in myeloid determinant genes. PU.1 ChIP-seq peak locations, scores and PU.1 motif scores. Data also show peak overlaps with publicly available datasets (0 = no peak present; 1 = peak present). For genes with >5 PU.1 peaks identified, the five highest-scoring peaks are shown. Only PU.1 ChIP-seq peaks overlapping with peaks in ≥2 publicly available datasets were considered.

SUPPLEMENTARY TABLE S3 Antibodies used in this study. List of all antibodies used in this study. Information includes clone, manufacturer and dilution.

SUPPLEMENTARY TABLE S4 Fluidigm primers used in this study. List of all primers including gene symbol, RefSeq accession number, and forward and reverse sequences.
