# Supplementary figures and images for: PU.1 is required to restrain myelopoiesis during chronic inflammatory stress
Source: Front Cell Dev Biol. 2023 Jun 26;11:1204160. doi: 10.3389/fcell.2023.1204160 (PMC10368259; doi:10.3389/fcell.2023.1204160)

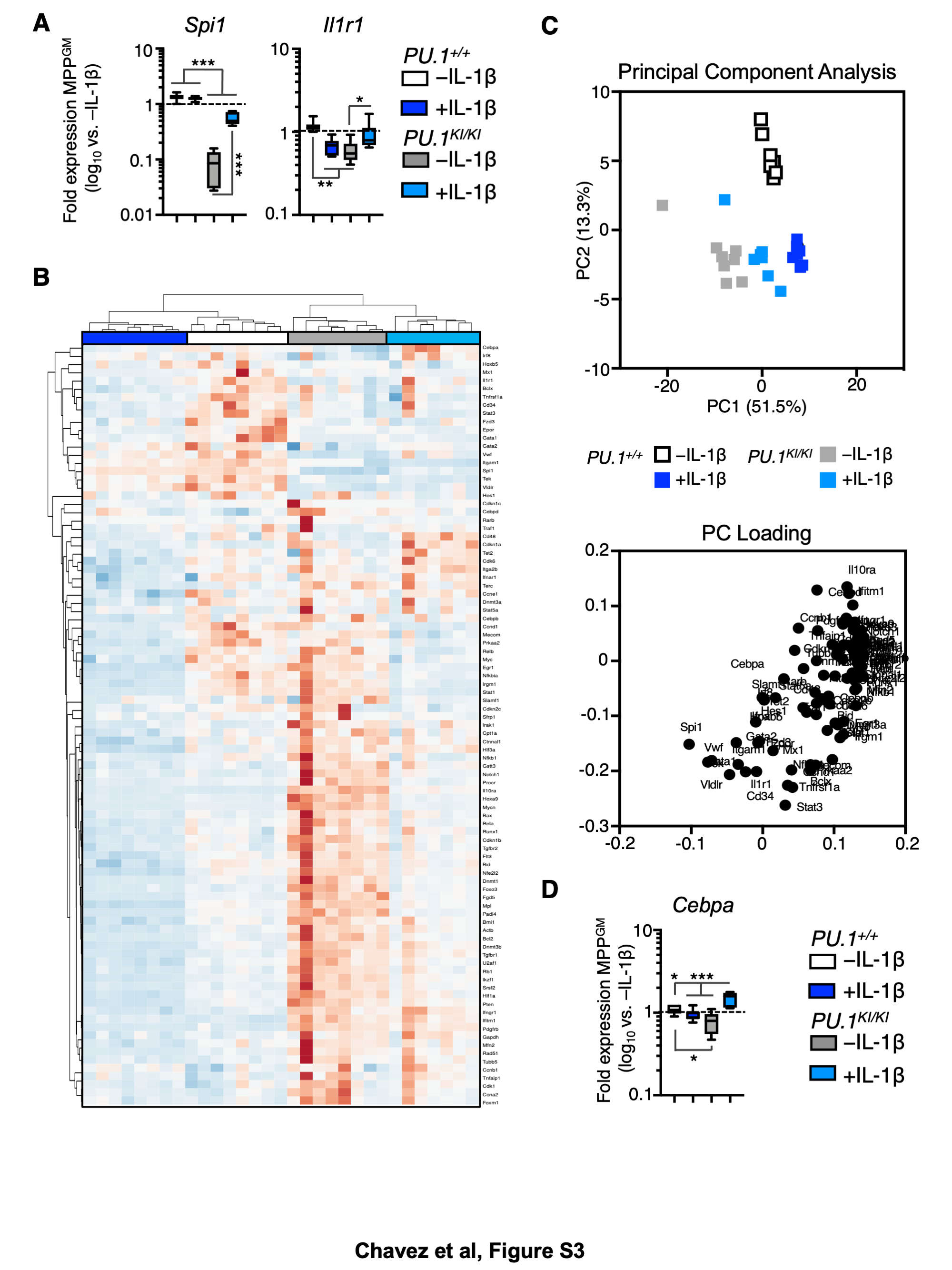

Supplement: Supplementary file 1 [file Image3.tiff]

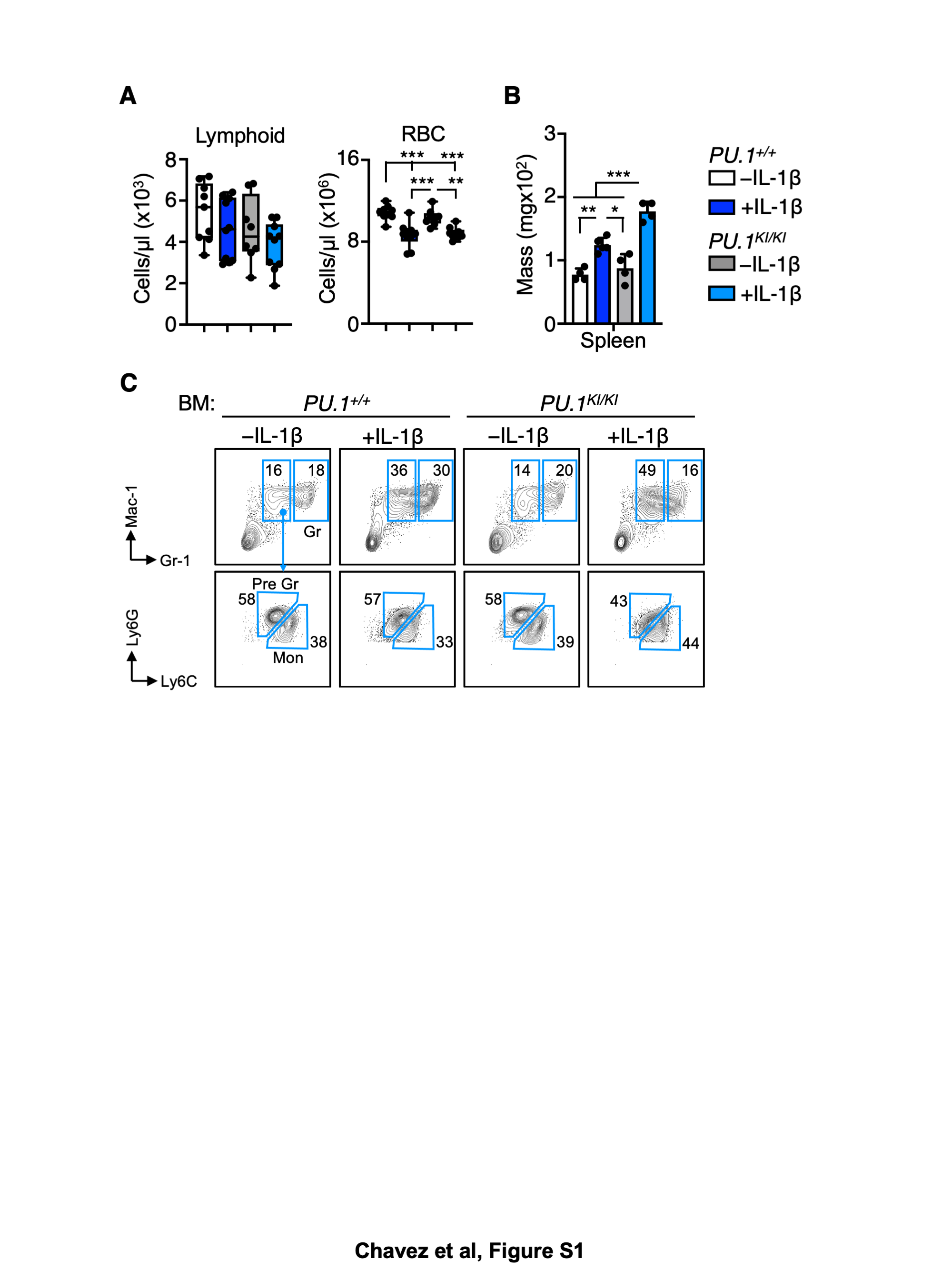

Supplement: Supplementary file 2 [file Image1.tiff]

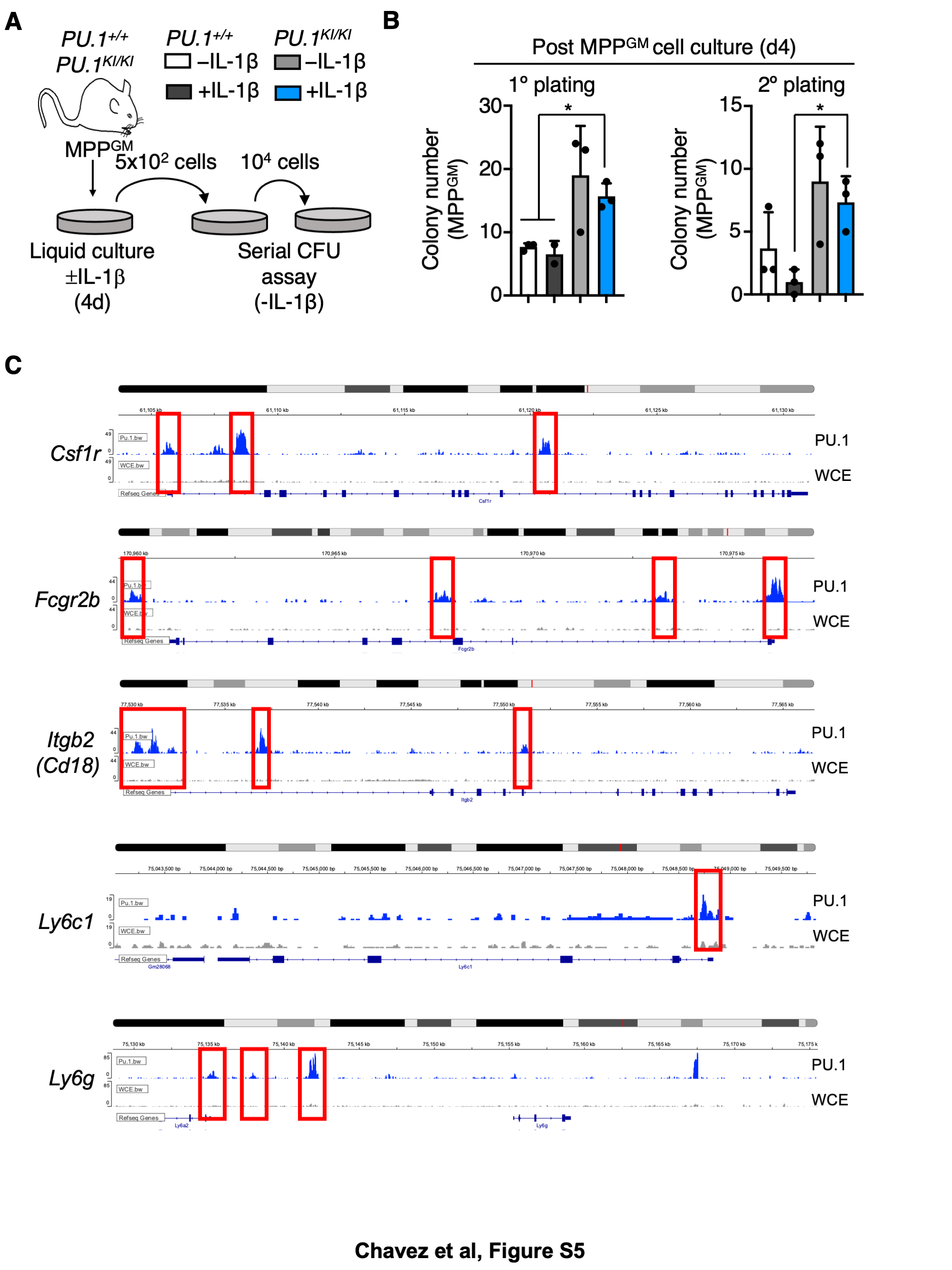

Supplement: Supplementary file 3 [file Image5.tiff]

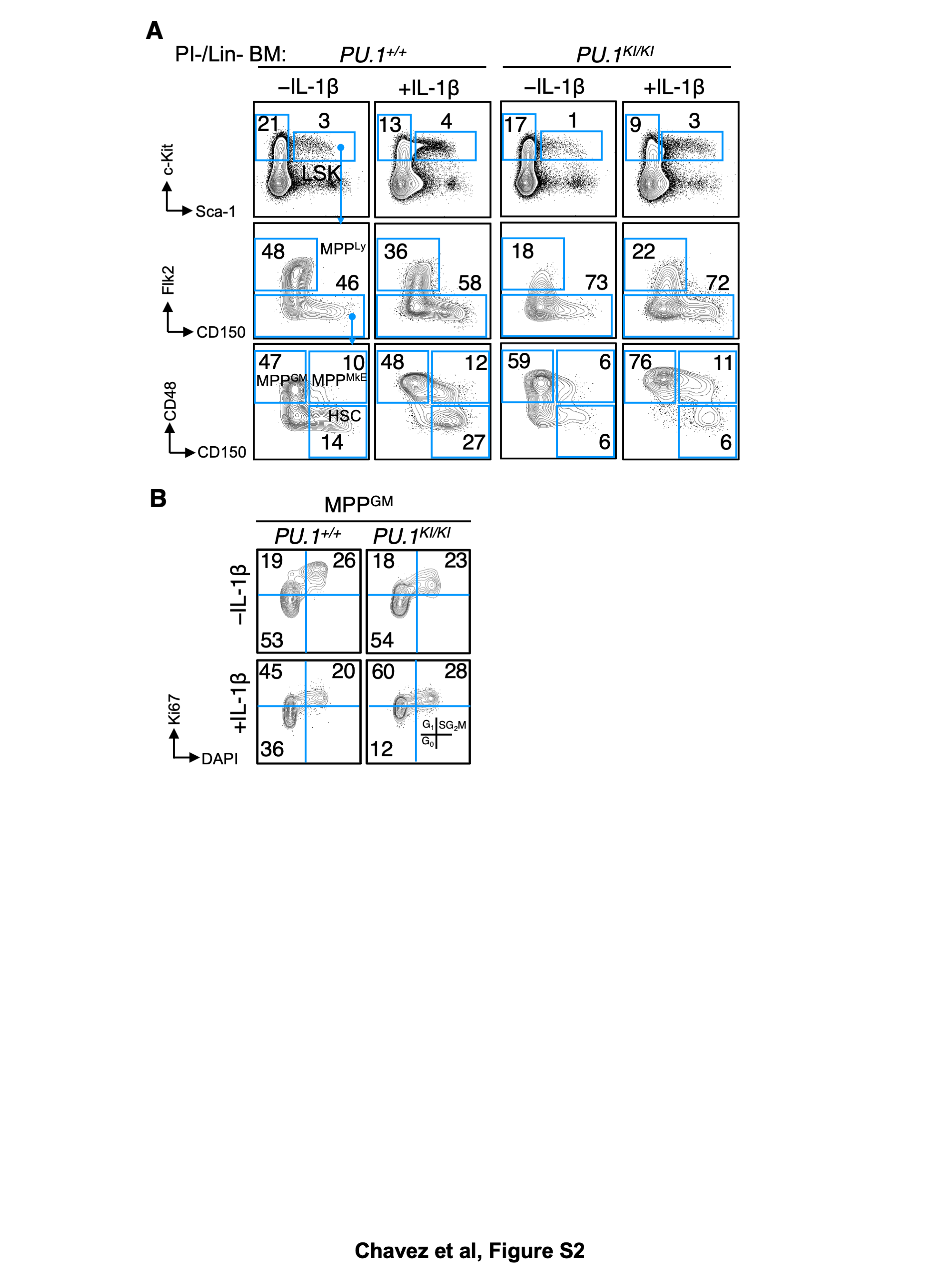

Supplement: Supplementary file 5 [file Image2.tiff]

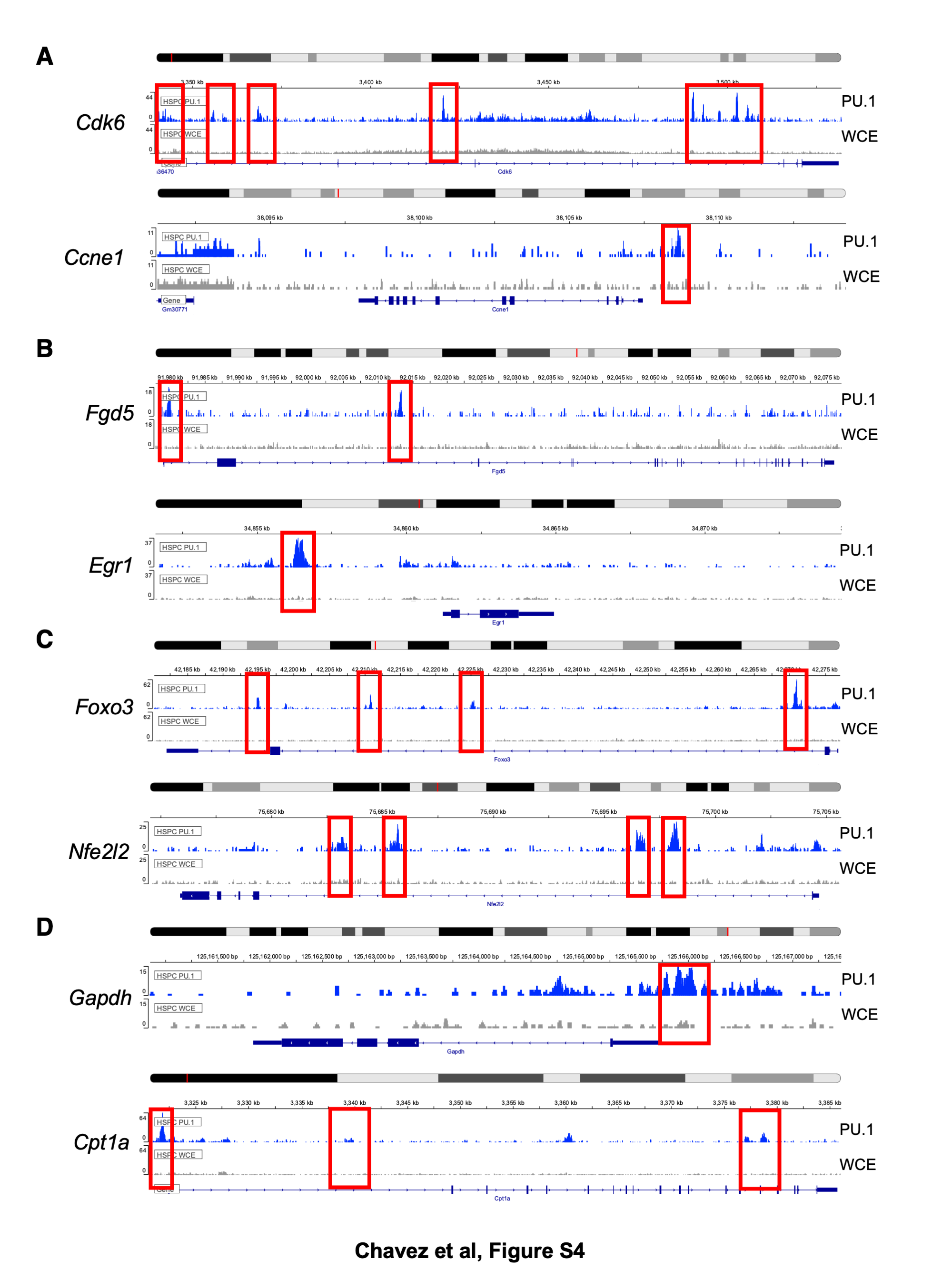

Supplement: Supplementary file 6 [file Image4.tiff]
